# Modulation of Astrocytes on Mode Selection of Neuron Firing Driven by Electromagnetic Induction

**DOI:** 10.1155/2020/8899577

**Published:** 2020-12-01

**Authors:** Zhongquan Gao, Zhixuan Yuan, Zuo Wang, Peihua Feng

**Affiliations:** ^1^School of Power and Energy Engineering, Xi'an Jiaotong University, Xi'an, Shaanxi 710049, China; ^2^State Key Laboratory for Strength and Vibration of Mechanical Structures, School of Aerospace Engineering, Xi'an Jiaotong University, Xi'an 710049, China

## Abstract

Both of astrocytes and electromagnetic induction are magnificent to modulate neuron firing by introducing feedback currents to membrane potential. An improved astro-neuron model considering both of the two factors is employed to investigate their different roles in modulation. The mixing mode, defined by combination of period bursting and depolarization blockage, characterizes the effect of astrocytes. Mixing mode and period bursting alternatively appear in parameter space with respect to the amplitude of feedback current on neuron from astrocyte modulation. However, magnetic flux obviously plays a role of neuron firing inhibition. It not only repels the mixing mode but also suppresses period bursting. The mixing mode becomes period bursting mode and even resting state when astrocytes are hyperexcitable. Abnormal activities of astrocytes are capable to induce depolarization blockage to compose the mixing mode together with bursting mode. But electromagnetic induction shows its strong ability of inhibition of neuron firing, which is also illustrated in the bifurcation diagram. Indeed, the combination of the two factors and appropriate choice of parameters show the great potential to control disorder of neuron firing like epilepsy.

## 1. Introduction

In order to understand complex physical and biochemical processes of signals generation and propagating in neural system, a powerful and basic model is proposed by Hodgkin and Huxley from a large number of experimental data on surface membrane of giant nerve of squids [1]. The Hodgkin-Huxley (HH) model explains successfully the switch between resting state and action potential from perspective of activation and inactivation of ion channels distributed in membrane of neuron. Different types of neuron firing like spiking and bursting can be captured by HH model, and it allows chaotic firing of neurons [2]. There are four variables in HH model, based on which, several simplified versions of model are developed to capture the main dynamics of neuron firing [3, 4]. Among them, ML (Morris and Lecar) model with three variables is considered to be elegant and simple to reveal dynamics of neuron firing [5].

Hundreds of studies have shown the validity and universality of ML model. Tsumoto proofs that the neuron firing can be adjusted by a single parameter via bifurcation theory [6]. The model can describe the oscillatory dynamics as a pacemaker triggered by Hopf bifurcation in muscle cells [7]. Ciszak distinguishes waking states and sleep states from perspective of neuron firing by ML model coupled with the spike time-dependent plasticity rule (STDP) [8]. Besides individual neuron firing activity, many researchers prefer to employ ML model to reveal dynamics of neuron spatiotemporal pattern in regular of complex neuronal networks. Hu proposes a complete electronic implementation of ML model for experimentally studying collective behaviors of neuronal network [9]. Wang discovers transition of pattern in a regular network consisting ML neurons coupled by chemical synapses [10]. And Feng further reveals the temporal property of pattern formation, which is characterized by phenomenon of critical slowing down [11].

Mode selection of neuron electrical activity is adjusted by different types of factors. It is believed that these factors, including ion channel noise [12], body temperature [13] and time delay [14, 15], are considered to trigger or suppress mental disorder via transferring neuron firing modes. More importantly, besides these factors, neuron firing is essentially modulated by glial cells. It is proofed experimentally that astrocytes not only support and nourish neurons but also involve in adjusting neuron information propagating among neuronal network through synapses [16–18]. Researchers illustrate the importance of calcium, glutamate, and ATP in chemical process of signal modulation between neurons by astrocytes. The glutamate, released from neurons to synaptic cleft, partially bounds to the receptors (mGluR) of the astrocytes membrane, leading generation of inositol 1,4,5-triphosphate (IP3) as the second messenger to trigger the release of calcium from endoplasmic reticulum. Stimulated by Ca2+ concentration increase, astrocytes release the glutamate to induce an additional inward current of neuron to influence information process. Suhita et al. propose a neuro-glial communication model based on HH model and predict spontaneous oscillations of neuron firing achieved by astrocyte modulation only [19]. Furthermore, astrocyte-neuron interaction is introduced into ML model to explore functional-based procedure with coexistence of excitatory and inhibitory synapses [20].

Furthermore, signal propagation in cortex achieved by electrical activity of neuron firing is affected greatly by electromagnetic environment inside skull according to Maxwell electromagnetic induction theorem [21]. It is hard to describe coupling of neuron firing and electromagnetic induction and radiation until invention of memristor [22, 23], which bridges neuron activity and electromagnetic flux via an equivalent current. Indeed, electromagnetic induction regulates neuron firing both in scale of individual neuron activity and in scale of spatiotemporal pattern formation of neuronal network. A model is established to study mode selection, and a practical circuit is designed to simulate neuron firing for this improved model [24]. Firing mode transition is achieved by magnetic flux [25], and it is further characterized as a route to chaos of neuron firing via frequent alternation of periodic and quasiperiodic motion [26, 27]. Parastesh et al. introduce discontinuous magnetic induction into neuron model [28]. Yuan numerically study the delay effect of external stimulation with consideration of magnetic flux [29]. Besides, many studies focus on collective behaviors in neuron network and pattern formation and stability loss process controlled by electromagnetic field effects [30]. In particular, Tian et al. consider magnetic flux in a chain-shape network of neurons and discovery the chimera state, a coexistence of synchronization and disorder state [31].

However, a comprehensive impact to neuron firing adjusted by astrocytes and exposed to electromagnetic environment does not explored deeply. In this paper, we address this problem by a neuro-glial system based on the ML model with consideration of electromagnetic induction.

Materials are listed as follows: a neuro-glial model modulated by electromagnetic field is described in Section 2. In Section 3, we disclose the key role of astrocytes leading special neuron modes, called mixing mode, when we do not take electromagnetic induction into account. Distribution of different firing modes in parameter space is provided. In Section 4, we compare both of astrocyte activities and electromagnetic induction and explore their different roles. Their dominated regions are illustrated in two-parameter space. Finally, conclusions are drawn in Section 5.

## 2. Governing Equation: Neuron-Astrocyte Model under Electromagnet Field

An improved ML model as a basic governing equation is selected to construct a model with consideration of electromagnetic induction and modulation of astrocytes. Membrane potential is determined not only by inward currents caused by Ca^2+^, delayed *K*^+^, and passive leak currents, respectively, but also by external stimulation, adjusted effect of astrocytes electromagnetic flux. The model is rewritten as follows [32],

(1) $$\begin{array}{c} C\frac{dv}{dt}=-g_{\text{Ca}}m_{\infty }\left(v-v_{\text{Ca}}\right)-g_{K}w\left(v-v_{K}\right)-g_{L}\left(v-v_{L}\right)+I_{\text{slow}}+I_{\mathrm{ext}}+I_{\text{ast}}+I_{\text{mag}}, \end{array}$$

(2) $$\begin{array}{c} \frac{dw}{dt}=\frac{\psi \left[w_{\infty }-w\right]}{\tau_{w}}, \end{array}$$

where

(3) $$\begin{array}{c} \begin{array}{c} m_{\infty }=0.5\left[1+\tanh\left(\frac{v-{\bar{v}}_{1}}{{\bar{v}}_{2}}\right)\right],\\ w_{\infty }=0.5\left[1+\tanh\left(\frac{v-{\bar{v}}_{3}}{{\bar{v}}_{4}}\right)\right],\\ \tau_{w}=\frac{1}{\cosh\left(v-{\bar{v}}_{3}/2{\bar{v}}_{4}\right)}, \end{array} \end{array}$$

(4) $$\begin{array}{c} \frac{{dI}_{\text{slow}}}{dt}=\epsilon \left(v^{*}-v-\alpha I_{\text{slow}}\right). \end{array}$$

*w* is the second variable of the model which represents the fraction of open channel of*K*^+^. *g*_Ca_, *g*_*K*_, and *g*_*L*_ are constant ion channel conductances. *m*_∞_, *w*_∞_, and *τ*_*w*_ are functions governing the ion channel dynamics and partially determine the neuron firing dynamics. *v*_*K*_, (*K* = 1, 2, 3, 4) are tuning parameters. *I*_slow_ gives birth to bursting behavior, and the parameters *ϵ* and *α* greatly modulate the rhythm and transition of neuron bursting.

It is assumed that IP_3_ is decided by the neurotransmitter release [*T*], and it controls Ca^2+^ concentration in cytoplasm. The change of calcium ion concentration consists of three parts: the Ca^2+^ flux from the endoplasmic reticulum (ER) to the cytosol *J*_channel_, the pump flux from cytoplasm to ER *J*_pump_, and the leakage flux from the ER to cytosol *J*_leak_. Activities of astrocytes, characterized by fluctuation of IP_3_ and Ca^2+^ concentration, are described as follows:

(5) $$\begin{array}{c} \frac{d\left[{\text{IP}}_{3}\right]}{dt}=\frac{1}{\tau_{{\text{IP}}_{3}}}\left({\left[{\text{IP}}_{3}\right]}^{*}-\left[{\text{IP}}_{3}\right]\right)+r_{{\text{IP}}_{3}}\left[T\right], \end{array}$$

(6) $$\begin{array}{c} \left[T\right]=\frac{1}{1+\exp\left(-\left(v-\theta_{s}\right)/\sigma_{s}\right)}, \end{array}$$

(7) $$\begin{array}{c} \frac{d\left[{\text{Ca}}^{2}+\right]}{dt}=-J_{\text{channel}}-J_{\text{pump}}-J_{\text{leak}}, \end{array}$$

(8) $$\begin{array}{c} \frac{dq}{dt}=\alpha_{q}\left(1-q\right)-\beta_{q}q, \end{array}$$

where

(9) $$\begin{array}{c} \begin{array}{c} J_{\text{channel}}=c_{1}v_{1}q_{\infty }^{3}n_{\infty }^{3}q^{3}\left(\left[{\text{Ca}}^{2+}\right]-{\left[{\text{Ca}}^{2+}\right]}_{\text{ER}}\right),\\ J_{\text{pump}}=\frac{v_{3}{\left[{\text{Ca}}^{2+}\right]}^{2}}{k_{3}^{2}+{\left[{\text{Ca}}^{2+}\right]}^{2}},\\ J_{\text{leak}}=c_{1}v_{2}\left(\left[{\text{Ca}}^{2+}\right]-{\left[{\text{Ca}}^{2+}\right]}_{\text{ER}}\right), \end{array} \end{array}$$

with

(10) $$\begin{array}{c} \begin{array}{c} q_{\infty }=\frac{\left[{\text{IP}}_{3}\right]}{\left[{\text{IP}}_{3}\right]+d_{1}},n_{\infty }=\frac{\left[{\text{Ca}}^{2+}\right]}{\left[{\text{Ca}}^{2+}\right]+d_{5}},\\ {\left[{\text{Ca}}^{2+}\right]}_{\text{ER}}=\frac{c_{0}-\left[{\text{Ca}}^{2+}\right]}{c_{1}},\\ \alpha_{q}=a_{2}d_{2}\frac{\left[{\text{IP}}_{3}\right]+d_{1}}{\left[{\text{IP}}_{3}\right]+d_{5}},\beta_{q}=a_{2}\left[{\text{Ca}}^{2+}\right]. \end{array} \end{array}$$

Amiri et al. propose a phenomenological model to describe feedback to neurons from astrocytes in a form of function *f*:

(11) $$\begin{array}{c} \frac{df}{dt}=\frac{-f}{\tau_{{\text{Ca}}^{2+}}}+\left(1-f\right)\kappa \Phi \left(\left[{\text{Ca}}^{2+}\right]-{\left[{\text{Ca}}^{2+}\right]}_{\text{th}}\right), \end{array}$$

where the Heaviside function *Φ* implies that gliotransmitter release is triggered by calcium inside the astrocytes only if its concentration is beyond a threshold [Ca^2+^]_th_. The additional currents induced by astrocytes added on the neuron are then described by

(12) $$\begin{array}{c} I_{\text{ast}}=\gamma f. \end{array}$$

As to the effect of electromagnetic induction, the corresponding current introduced into the membrane potential is realized by memristor which bridges magnetic flux across the membrane and potential. *ρ*(*ϕ*) is the memory conductance of external current caused by magnetic flux, which is usually chosen as a nonlinear form. In summary, the model governing the magnetic induction is described as follows:

(13) $$\begin{array}{c} \begin{array}{c} I_{\text{mag}}=-k_{1}\rho \left(\phi \right)V,\\ \frac{d\phi }{dt}=V-k_{2}\phi ,\\ \rho \left(\phi \right)=\alpha +3{\beta \phi }^{2}, \end{array} \end{array}$$

where *k*_1_ measures the feedback strength from electromagnetic induction and *k*_2_ is natural decay rate in the assumed homogeneous medium. The values of parameters are listed in Table.  1.

## 3. Mixing Neuron Firing Mode Selection Modulated by Astrocytes without Magnetic Flux

The parameter *r*_IP_3__ reflects the activities of astrocytes corresponding to neuron firing by determining the production of IP_3_ in astrocyte cytoplasm triggered by neuron spikes. When membrane potential increases beyond a threshold *θ*_*s*_, the astrocytes begin to release IP_3_ to stimulate Ca^2+^ concentration enhancement. It has been proofed that *r*_IP_3__ production can reduce threshold of spontaneous oscillation of neuron firing, and when *r*_IP_3__ is large enough, the neuron could oscillate without any external stimulation [19]. It is reasonable that the results are considered as an evidence epilepsy can also be induced by abnormal astrocyte activities beside neuron firing disorder.

**Figure 1:**
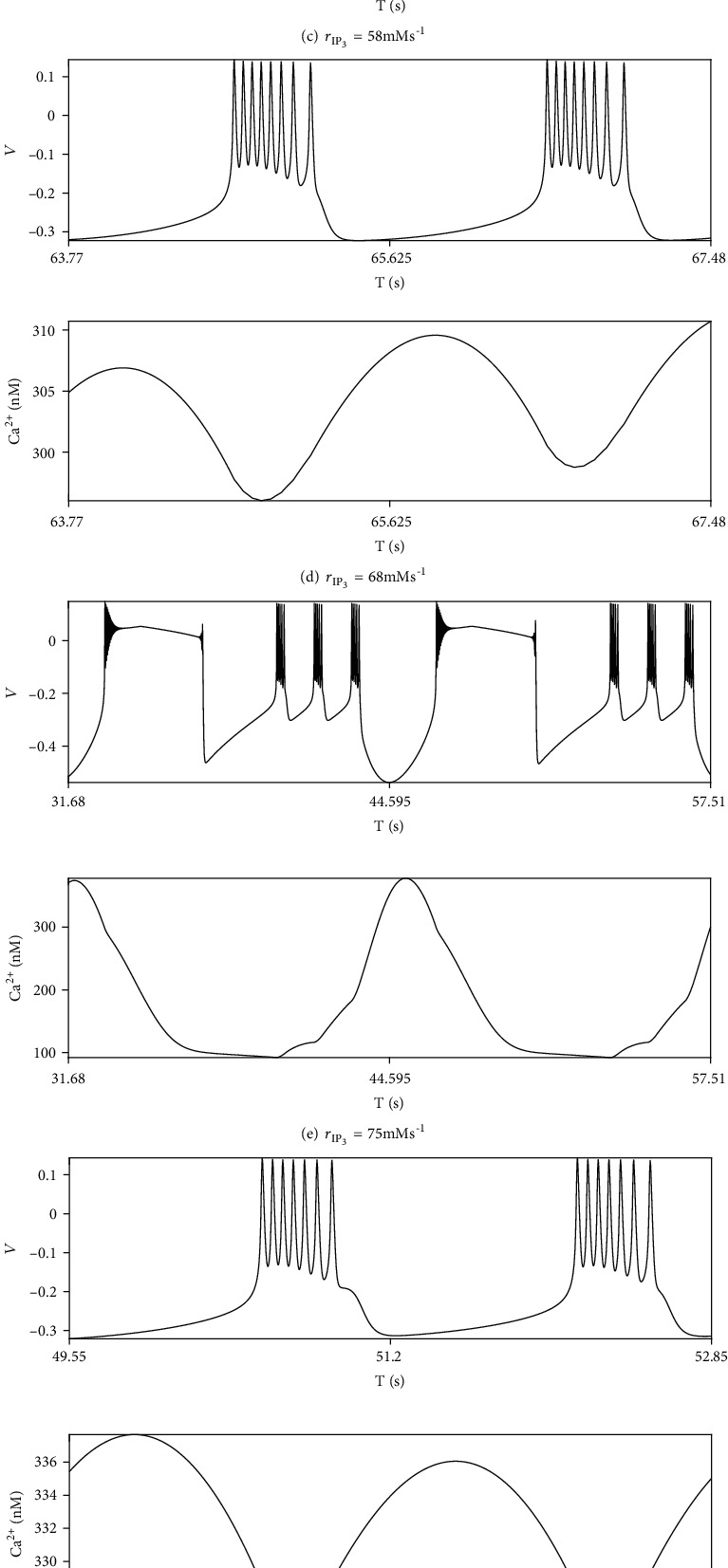
The neuron firing modes and Ca^2+^ activities with no electromagnetic induction under different *r*_IP_3__.

In order to highlight the role of astrocytes in our study, we first illustrate the dynamic role of IP_3_ production in modulating neuron firing in ML model without magnetic induction into account (*k*_1_ = 0). We provide the neuron spike with Ca^2+^ oscillation in a large span of parameter *r*_IP_3__ in Figure 1. The motion of neuron firing remains period-4 bursting until *r*_IP_3__ is greater than 40, at which the system transfers to period-9 bursting (Figures 1(a) and 1(b)). When *r*_IP_3__ continually increases a little, the motion of system enters a stage of alternation between periodic busting and depolarization blockage. In blockage, the membrane potential remains in a high level for a while and a spike cannot be sustained which is a typical abnormal firing mode in electrographic seizures [33]. Similarly, in the following interval of *r*_IP_3__, the motion switches back and forth between pure periodic bursting and the stage of alternation (between bursting and depolarization blockage). In Figures 1(c), 1(e), and 1(g), *r*_IP_3__ locates in the intervals where motions of periodic bursting are interrupted by depolarization blockages. And the number of bursting between every two adjacent depolarization blockages decreases. The rest intervals represent period-4, period-9, period-8, period-6, and period-7 firing modes, respectively.

**Figure 2:**
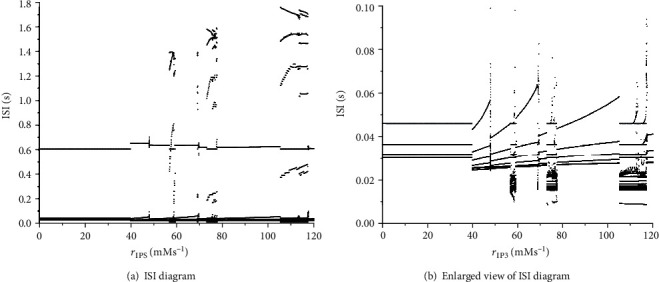
The ISI diagram respect to *r*_IP_3__ and its enlarged view.

Interspike interval (ISI) diagram also confirms the transition from the alternation of two firing modes to depolarization blockage (see Figure 2). The intervals where the ISI burst out represent blockage and periodic bursting, because only in this mode can ISI reach every high values and occupy a very large span. The rest of them, denoted in the partial enlarged view, are firing modes of periodic bursting with different spike numbers.

**Figure 3:**
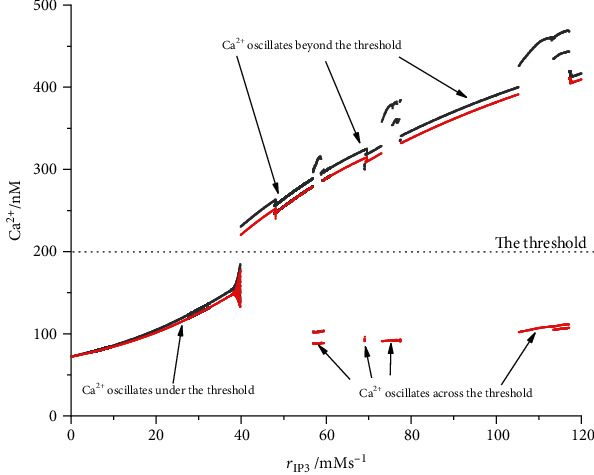
Crests and troughs of the oscillation of Ca^2+^ concentration respect to *r*_IP_3__. Black dots denote the crest, and red ones are troughs of the oscillation. The dashed line means the threshold.

Compared to the evolution of Ca^2+^, we confirm that every time when concentration penetrates downward the threshold, the end of depolarization blockage is discovered. When Ca^2+^ concentration is beneath the threshold, the current denoting the astrocyte activities turns off. On contrary, if the minimum of Ca^2+^ concentration is beyond the threshold, the current always turns on. Both of the two cases correspond to periodic bursting. However, as threshold locates inside the span of Ca^2+^ oscillation, the current alternatively and continuously turns on and off. Only in this case is the mixing firing mode observed in which depolarization blockage and periodic bursting appears alternatively. We plot the crests and troughs of the oscillation of Ca^2+^ concentration compared to the threshold to represent the three cases in Figure 3.

**Figure 4:**
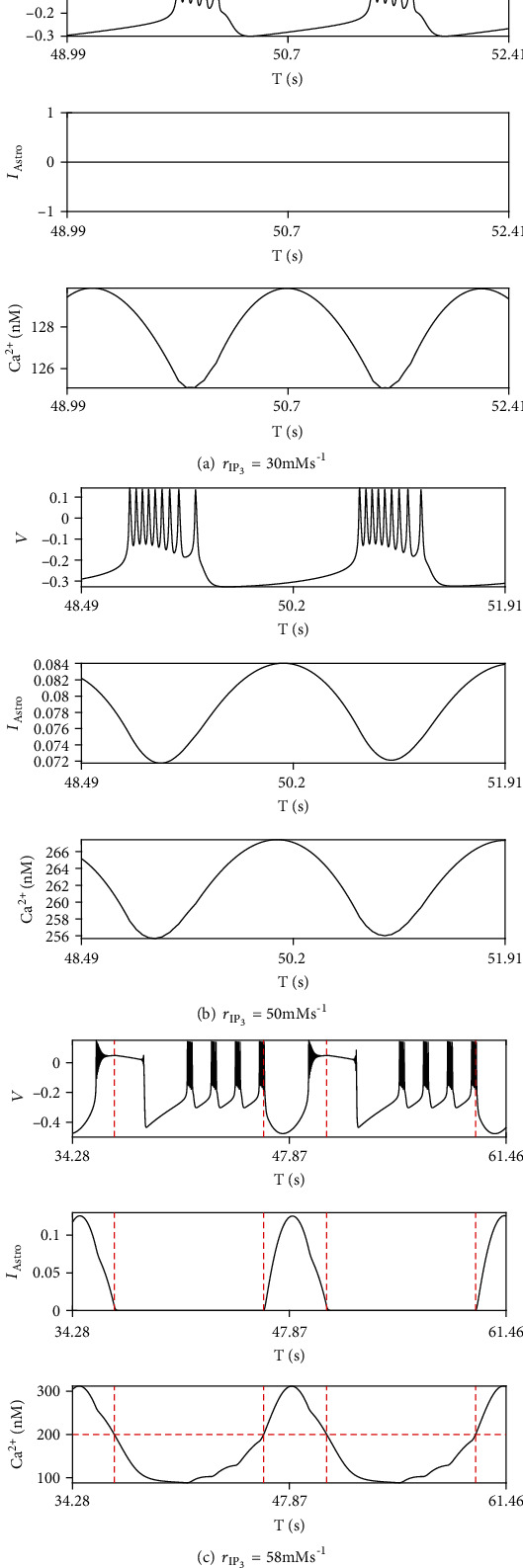
Membrane potential compared with feedback current and Ca^2+^ activities in (a) Ca^2+^ is under threshold (*r*_IP_3__ = 30mMs^‐1^), (b) Ca^2+^ oscillates across threshold (*r*_IP_3__ = 50mMs^‐1^), and (c) Ca^2+^ oscillates above the threshold (*r*_IP_3__ = 58mMs^‐1^).

The current applied to the membrane potential from astrocytes *I*_ast_ definitely plays an important role in mode selection of neuron firing. In order to further specify the magnificence of astrocytes, membrane potential compared with astrocyte feedback current *I*_ast_ in three type of cases at different *r*_IP_3__, (i) Ca^2+^ is under the threshold (*r*_IP_3__ = 30mMs^‐1^), (ii) Ca^2+^ oscillates across the threshold (*r*_IP_3__ = 50mMs^‐1^), and (iii) Ca^2+^ oscillates above the threshold (*r*_IP_3__ = 58mMs^‐1^). *I*_ast_ keeps zero, and Ca^2+^ oscillates under the threshold (Figure 4(a)), which means that the astrocyte do not release gliotransmitters and is absent in modulating neuron firing. When Ca^2+^ oscillates beyond the threshold, *I*_ast_ begins to oscillate with a very small fluctuation of amplitude, and neuron remains in bursting state (Figure 4(b)). And it seems that the number of spikes in every periodic bursting in this case is more than that when Ca^2+^ is under the threshold. In the mixing mode Ca^2+^ oscillates in a large span of amplitude as *r*_IP_3__ = 58mMs^‐1^ (Figure 4(c)). *I*_ast_ shrinks to zero accompanied with that Ca^2+^ drops to the threshold. At the same time, the oscillation of membrane potential attenuates quickly and remains in a high level until it falls to a negative value. During the interval in which *I*_ast_ keeps zero (Ca^2+^ is under the threshold), periodic bursting generates and is interrupted by that Ca^2+^ increases and passes the threshold to trigger the release of gliotransmitters.

## 4. Neuron Firing Modulated Both with Astrocytes and under Electromagnetic Flux

**Figure 5:**
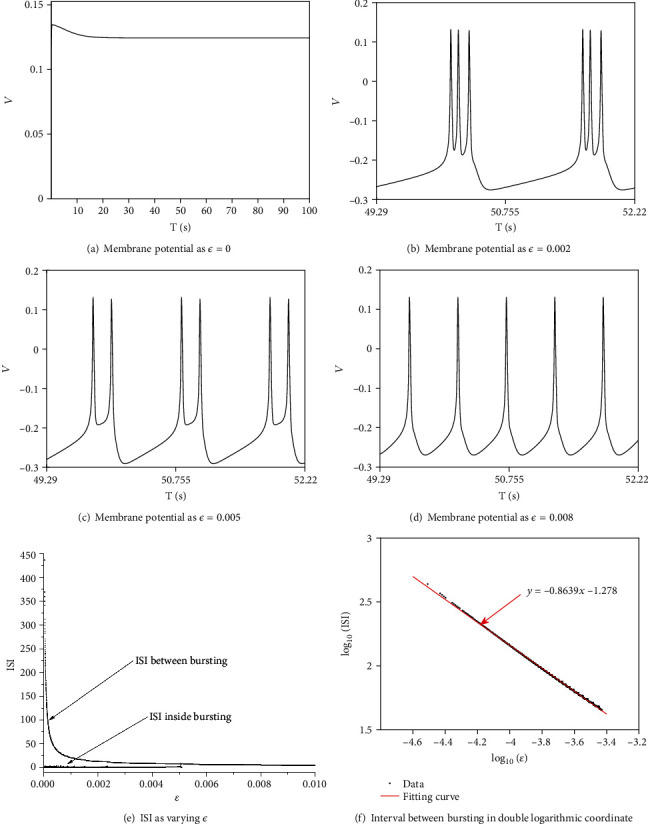
Membrane potential and ISI diagram varying *ϵ* with electromagnetic induction (*k*_1_ = 0.5) considered but without astrocytes (*r*_IP_3__ = 0mMs^‐1^).

Before elucidating the role of electromagnetic induction, we specify the effect of *I*_slow_ in generating and controlling neuron firing. Periodic bursting vanishes, and neuron keeps in resting state when there is no *I*_slow_ (*ϵ* = 0, Figure 5(a)). Periodic bursting or oscillation exists when *ϵ* is a small positive value. Span of interspike interval between every cluster decreases rapidly accompanied with the fact that spike number in every period also decreases, when we increase *ϵ*. Periodic bursting becomes periodic oscillation as *ϵ* passes a critical value. The scenario is illustrated by membrane potential at different values of *ϵ* (Figures 5(b)–5(d)). The upper branch in ISI diagram (Figure 5(e)) denotes the intervals between clusters, and lower one denotes the intervals between spikes inside the clusters. The two branches converge to one branch at the critical value of *ϵ* where periodic oscillation replaces the periodic bursting. ISI between bursting, denoted by upper branch, shrinks in the power law, which means that span of ISI and the parameter *ϵ* satisfy ISI = *A*∗*e*^−*λ*^. The truth is illustrated by a line in double logarithmic coordinate with *λ* = 0.8639. In this study, *ϵ* is kept in 0.002 in order to guarantee the existence of periodic bursting.

Beside feedback current from astrocytes, electromagnetic induction also plays a key role in modulating neuron firing. In the model we improved, *k*_1_ is the most important parameter to measure the magnitude of feedback current. We select three different cases to elucidate the role of electromagnetic induction: (a) only induction adjusts neuron firing *r*_IP_3__ = 0mMs^‐1^, (b) we start from periodic clustering as *r*_IP_3__ = 80mMs^‐1^ and vary *k*_1_, and (c) *r*_IP_3__ = 100mMs^‐1^ is chosen at which alternation of periodic clustering and depolarization blockage occurs at *k*_1_ = 0.

**Figure 6:**
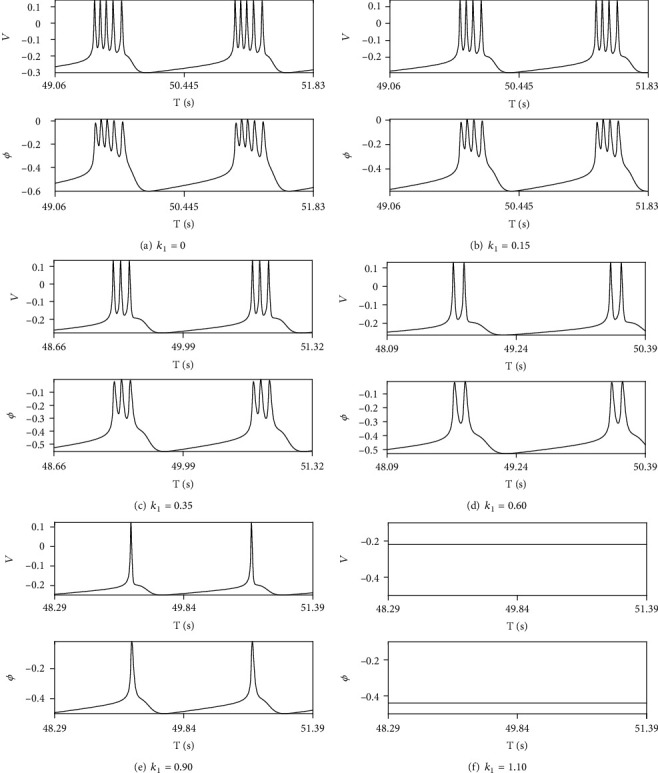
The neuron firing modes and magnetic flux *ϕ* without astrocyte activities.

Without oscillation of Ca^2+^ concentration, depolarization blockage cannot exist in firing mode, leading that only periodic bursting is left as varying amplitude of feedback current *k*_1_. Transition from periodic bursting to resting state is achieved by increasing *k*_1_ with setting *r*_IP_3__ = 0mMs^‐1^ (see Figure 6). Number of spikes during one period decreases leading that period-5, period-4, period-3, period-2, period-1 bursting, and resting state are displayed in turn.

**Figure 7:**
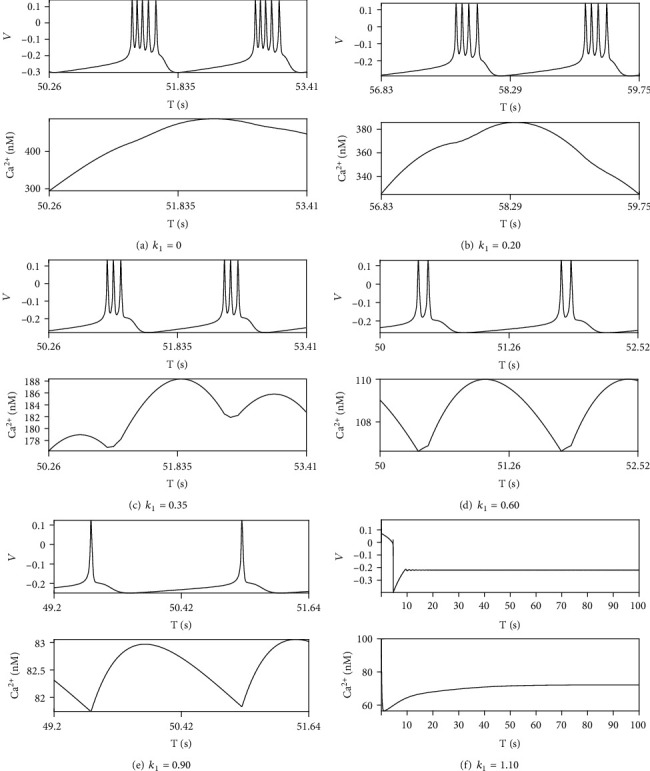
The neuron firing modes and Ca^2+^ activities as *r*_IP_3__ = 80mMs^‐1^.

There is no mixing mode when *r*_IP_3__ = 80mMs^‐1^, see Figure 7. Ca^2+^ concentration oscillates either beyond the threshold (Figures 7(a) and 7(b)) or beneath the threshold (Figures 7(c)–7(e)). Ca^2+^ concentration does not oscillate around the threshold which guarantee that depolarization blockage cannot be appear in firing modes. Resting firing dominates the firing mode as *k*_1_ is greater than 1 (Figure 7(f)).

**Figure 8:**
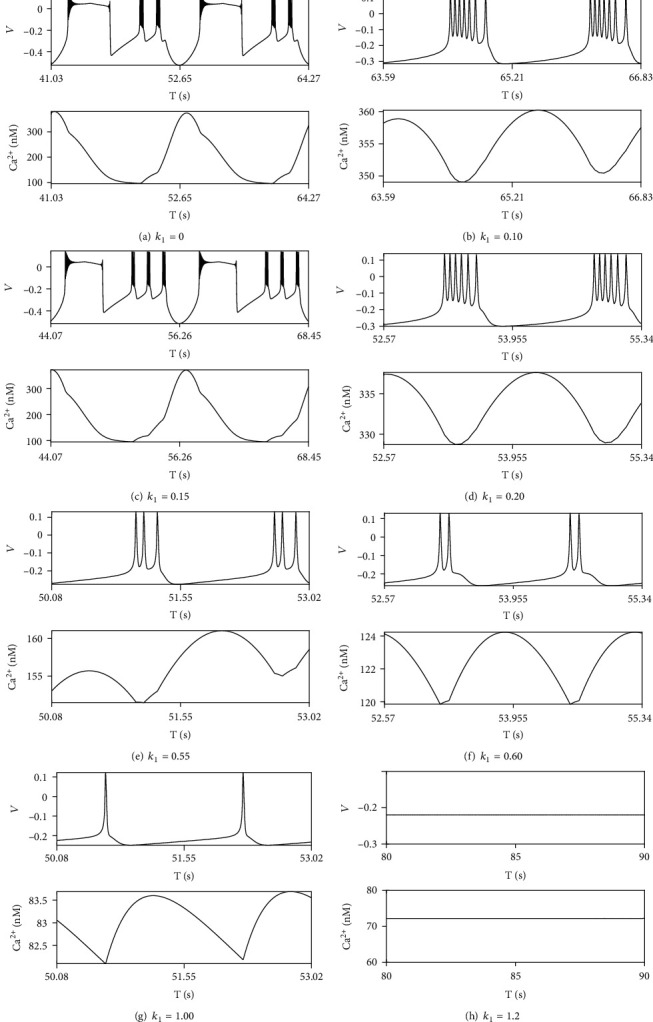
The neuron firing modes and Ca^2+^ activities as *r*_IP_3__ = 100mMs^‐1^.

With *r*_IP_3__ = 100mMs^‐1^, different neuron firing modes represent in Figure 8 as varying *k*_1_. Mixing mode is observed when *k*_1_ = 0, but it suddenly vanishes when *k*_1_ is little bit more than 0. Mixing modes cannot be discovered only except a small interval 0.14 < *k*_1_ < 0.17, leading periodic bursting in firing mode. Number of spikes in one period decreases with respect to *k*_1_. Period-7 bursting becomes period-6 as *k*_1_ becomes 0.20 from 0.10; then, it reduces to period-3, period-2, and at last period-1 when *k*_1_ gets approach to 1.0. When *k*_1_ is greater than 1, spike neuron firing disappears, and only oscillation with very small amplitude under threshold exists.

**Figure 9:**
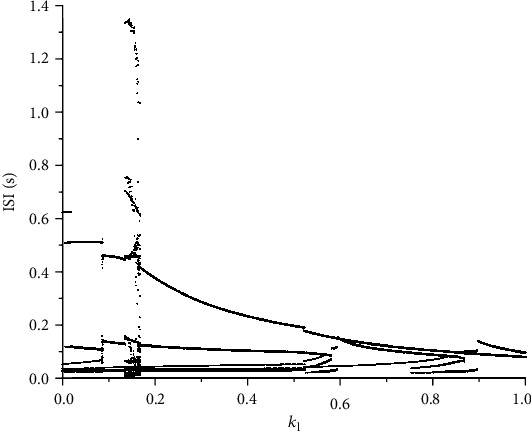
The ISI diagram respect to *k*_1_ with *r*_IP_3__ = 100mMs^‐1^.

ISI diagram also illustrates the same statement by reducing span of ISI values with increasing *k*_1_ (see Figure 9). Obviously, the current from electromagnetic induction introduced into membrane potential play a key role of suppressing spike neuron firing. Mixing mode is rarely observed in neuron firing, and periodic bursting vanishes, and firing mode becomes resting state when amplitude of current is large enough.

**Figure 10:**
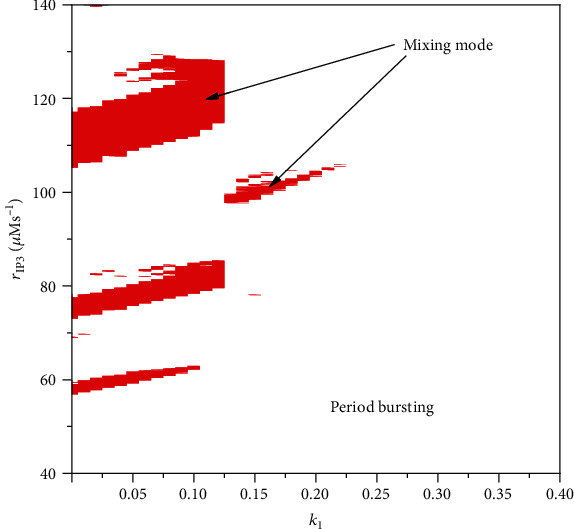
Regions dominated by mixing mode or periodic bursting in parameter space. Red regions denote the mixing mode, and white region is periodic bursting.

We also provide the two-parameter space to show distribution of mixing mode, which illustrate the great ability that electromagnetic induction suppresses the depolarization blockage or mixing mode. As illustrated in Figure 10, mixing mode only exists in some isolate parameter regions when *k*_1_ < 0.22. The rest of regions are dominated by periodic bursting and resting state.

**Figure 11:**
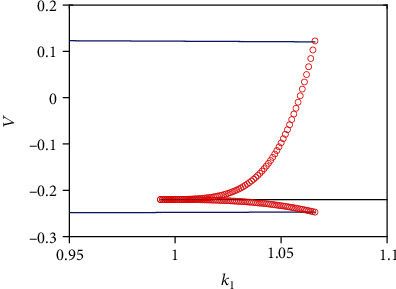
Bifurcation diagram as varying amplitude of feedback current from electromagnetic induction *k*_1_.

In order to further specify the inhibition role of electromagnetic induction, we provide the bifurcation diagram with *k*_1_ as the control parameter (Figure 11). The limit circle (denoted by blue lines) becomes a stable focus (denoted by black line) via saddle-node bifurcation. This transition means that violent oscillations dominated by a limit circle when *k*_1_ is small enough, degenerate into small oscillations or resting states when the motion is in the basin of stable focus as increasing *k*_1_.

## 5. Conclusions and Prospects

In this paper, we numerically explore scenario of neuron firing mode selection among mixing mode, periodic bursting, and resting state. Mixing mode is an alternation stage between periodic bursting and depolarization blockage. Depolarization blockage appears only when Ca^2+^ concentration goes up through the threshold and ends when Ca^2+^ concentration penetrates down through the threshold. Therefore, mixing mode dominates the firing mode only when concentration of Ca^2+^ oscillate across value of threshold.

When there is no electromagnetic induction adjusting neuron firing mode, introduction of astrocytes could induce depolarization blockage into neuron firing, with combination of periodic bursting to compose mixing mode. Mixing mode and periodic bursting distribute alternatively in the parameter space. With respect to *r*_IP_3__, the interval between every two adjacent depolarization blockages shrinks, and the number of periodic clustering firing decreases. But when the amplitude of feedback current is large enough, mixing mode cannot be observed in neuron firing because Ca^2+^ concentration is beyond the threshold.

The role of slow current is also studied. The number of spikes in one period decreases, and span of ISI between clusters shrinks in a power law when increasing the amplitude of slow current *ϵ* and finally brings periodic bursting into periodic oscillation. Electromagnetic induction plays an important role of suppressing neuron firing. As amplitude of feedback current *k*_1_ increasing, mixing mode shrinks gradually, and *k*_1_ is greater than 1, the mixing mode is completely vanishes. Distribution in two-parameter space also indicates the same statement. The inhibition role of electromagnetic field is explained by saddle-node bifurcation which brings violent oscillations into small ones or resting state.

Our research focuses on neuron firing modes transition and dynamical properties of them in the improved ML model, with consideration of both astrocytes and electromagnetic induction. We compare different role of them and study numerically their effects of modulation on neuron firing. Therefore, the results are of great potential significance on control strategies of mode selection by controlling amplitude of feedback currents introduced by both of the two factors and even on therapy of refractory diseases including suppressing epilepsy and realizing defibrillation.

## Figures and Tables

**Table 1:** Temperature and wildlife count in the three areas covered by the study.

Parameter	Value	Unit	Parameter	Value	Unit
*v*_1_	6	s^‐1^	[IP_3_]^∗^	0.16	*μ*M
*c*_1_	0.185	–	*r*_IP_3__	7.2	mMs^‐1^
*g*_Ca_	1	–	*v*_4_	0.145	–
*v*_Ca_	1	–	*v*_2_	0.11	s^‐1^
*v*_3_	0.9	*μ*Ms^‐1^	*g*_*K*_	2	–
*v*_*K*_	-0.7	–	*θ*_*s*_	0.2	–
*g*_Ca_	1	–	*d*_3_	0.94	*μ*M
*d*_1_	0.13	*μ*M	*d*_2_	1.05	*μ*M
*g*_*L*_	0.5	–	*σ*_*s*_	0.02	–
*v*_*L*_	-0.5	–	[Ca^2+^]_th_	200	nM
*c*_0_	-2	*μ*M	*d*_5_	0.082	*μ*M
*a*_2_	0.2	*μ*Ms^‐1^	*ψ*	1.15	–
${\bar{v}}_{1}$	-0.01	–	*κ*	0.5	s^‐1^
*τ*_IP_3__	7	s	*τ*_Ca^2+^_	6	s
${\bar{v}}_{2}$	0.15	–	${\bar{v}}_{3}$	0.1	–
${\bar{v}}_{4}$	0.145	–	*k*_1_	0.01	–
*k*_2_	0.5	–	*α*	0.1	–
*β*	0.1	–			

## Data Availability

The data used to support the findings of this study are available from the corresponding author upon request.
